# Effect of bladder filling on doses to prostate and organs at risk: a treatment planning study

**DOI:** 10.1120/jacmp.v8i1.2286

**Published:** 2007-02-28

**Authors:** Vitali Moiseenko, Mitchell Liu, Sarah Kristensen, Gerald Gelowitz, Eric Berthelet

**Affiliations:** ^1^ Fraser Valley Centre British Columbia Cancer Agency Surrey British Columbia Canada; ^2^ Vancouver Island Centre British Columbia Cancer Agency British Columbia Canada

**Keywords:** Prostate cancer, organ motion, radiotherapy, bladder filling

## Abstract

In the present study, we aimed to evaluate effects of bladder filling on dose–volume distributions for bladder, rectum, planning target volume (PTV), and prostate in radiation therapy of prostate cancer. Patients (n=21) were scanned with a full bladder, and after 1 hour, having been allowed to void, with an empty bladder. Radiotherapy plans were generated using a four‐field box technique and dose of 70 Gy in 35 fractions. First, plans obtained for full‐ and empty‐bladder scans were compared. Second, situations in which a patient was planned on full bladder but was treated on empty bladder, and vice versa, were simulated, assuming that patients were aligned to external tattoos. Doses to the prostate [equivalent uniform dose (EUD)], bladder and rectum [effective dose $\left(D_{\text{eff}}\right)$], and normal tissue complication probability (NTCP) were compared. Dose to the small bowel was examined. Mean bladder volume was 354.3 cm^3^ when full and 118.2 cm^3^ when empty. Median prostate EUD was 70 Gy for plans based on full‐ and empty‐bladder scans alike. The median rectal $D_{\text{eff}}$ was 55.6 Gy for full‐bladder anatomy and 56.8 Gy for empty‐bladder anatomy, and the corresponding bladder $D_{\text{eff}}$ was 29.0 Gy and 49.3 Gy respectively. In 1 patient, part of the small bowel (7.5 cm^3^) received more than 50 Gy with full‐bladder anatomy, and in 6 patients, part $\left(2.5\;{\text{cm}}^{3}-30\;{\text{cm}}^{3}\right)$ received more than 50 Gy with empty‐bladder anatomy. Bladder filling had no significant impact on prostate EUD or rectal $D_{\text{eff}}$. A minimal volume of the small bowel received more than 50 Gy in both groups, which is below dose tolerance. The bladder $D_{\text{eff}}$ was higher with empty‐bladder anatomy; however, the predicted complication rates were clinically insignificant. When the multileaf collimator pattern was applied in reverse, substantial underdosing of the planning target volume (PTV) was observed, particularly for patients with prostate shifts in excess of 0.5 cm in any one direction. However, the prostate shifts showed no correlation with bladder filling, and therefore the PTV underdosing also cannot be related to bladder filling. For some patients, bladder dose–volume constraints were not fulfilled in the worst‐case scenario—that is, when a patient planned with full bladder consistently arrived for treatment with an empty bladder.

PACS numbers: 87.53.‐j, 87.53.Kn, 87.53.Tf

## I. INTRODUCTION

Currently, most cancer centers use full‐bladder protocols for radiation therapy of patients with prostate cancer. The rationale for this choice is well established, being based on better sparing of the bladder and small bowel. Currently available technologies for conformal delivery of radiation to the target, and specifically, for conformal avoidance, may make the advantages of full‐bladder treatment less pronounced. In addition, data about the reproducibility of patient perception of a full bladder are lacking. However, it is acknowledged that, through the course of radiation therapy, bladder filling may vary in a systematic manner.^(1)^ Not uncommonly, either because of advanced age or irritating urinary symptoms, prostate cancer patients find it difficult to maintain a full bladder during radiotherapy. Filling of the bladder may also significantly affect prostate position and have a negative impact on the accuracy of radiotherapy.^(^ ^2^ ^–^ ^5^ ^)^ Empty‐bladder treatment has therefore been advocated in patients who require radiation therapy to the prostate alone. This approach provides better patient comfort and potentially better reproducibility. Empty‐bladder protocols for radiation therapy of the prostate are coming into use, and the reported bladder toxicity is low so far.^(^ ^6^ ^,^ ^7^ ^)^

Evaluation of treatment plans is based on proper coverage of target volumes by the isodose lines of choice and sparing of organs at risk (OARs). In prostate radiation therapy, a common criterion is that the planning target volume (PTV) be fully contained within the 95% isodose surface. The OARs include femurs, bladder, and rectum. The rectum is often the dose‐limiting organ. Dose–volume criteria and constraints have been established to keep toxicity at acceptable levels. Specifically, for prostate radiation therapy, Radiation Therapy Oncology Group criteria (RTOG P0126) are commonly used.

Data sets for conformal therapy outcome with full and empty bladder are both becoming available, but direct comparison is often complicated by patient selection, treatment protocol, prescribed dose, and plan acceptance criteria. Data on the effect of bladder filling on prostate motion in prone^(^ ^2^ ^,^ ^5^ ^)^ and supine^(^ ^8^ ^–^ ^10^ ^)^ positions are limited. The recent study by Pinkawa et al.^(10)^ specifically addressed dose–volume distributions as a function of bladder filling with prostate patients who underwent computed tomography (CT) scanning before radiation therapy and at 4 and 8 weeks of radiation therapy. The authors noted larger variations in full‐bladder volume as compared with empty‐bladder volume as treatment progressed. However, that variation did not affect prostate position.

Ideally, bladder‐filling data as it pertains to prostate cancer radiation therapy should be obtained in a controlled environment that eliminates factors other than bladder filling. A study specifically designed to evaluate how dose–volume distributions in the PTV, the clinical target volume (CTV, defined as the prostate only), and the OARs change depending on bladder filling can therefore provide justification for conformal‐field treatment on an empty bladder. In the present paper, we report the effects of bladder filling on dose distributions for CTV, PTV, bladder, rectum, and small bowel with prostate patients first scanned on a full bladder and then on an empty bladder after 1 hour was allowed for voiding.

## II. METHODS

### A. CT scanning

We enrolled 21 patients aged between 60 and 80 years who received radiation therapy for prostate cancer at our cancer center. All patients gave consent to participate in the study.

**Figure 1:**
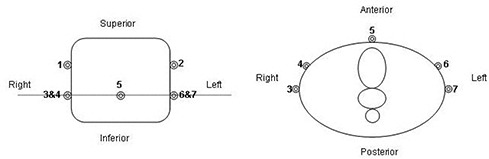
Illustration of fiducial marker placement: coronal view (left) and axial view (right).

We used a PQ 2000 scanner (Philips Medical Systems, Andover, MA) to obtain full‐ and empty‐bladder CT scans for each patient. Patients were positioned supine with a foam cushion for knee support and a Perspex form for ankle support. The first scan was obtained on a full bladder, patients having been asked to drink 2–3 glasses of fluid (500–750 mL) at least 30 minutes before the scan. Seven fiducial markers were placed on each patient to ensure consistent setups during the full‐ and empty‐bladder scans (Fig. 1). Markers 3 and 7 were placed at the level of the right and left greater trochanters; markers 4 and 6 were placed 4.0 cm anterior to markers 3 and 7, with marker 5 being placed at the anterior midline in the same plane; and markers 1 and 2 were placed on the right and left, 5 cm superior to markers 3, 7, and 5. Scanning limits were set from the level of L2/L3 to 4 cm inferior to the ischial tuberosities, using a 0.5‐cm slice index and thickness. Contouring and registration were performed using Philips AcQsim software. The prostate was contoured on the full‐bladder scan while the patient remained on the CT couch. The virtual simulation software was used to determine the prostate center of mass, and based on the resulting coordinates, the patient was tattooed using the laser system. Following the full‐bladder scan, the patients were given 1 hour to void before the empty‐bladder CT scan was obtained. The seven external fiducial markers were then used to register and evaluate the two scans for accuracy.

The entire bladder, rectum (from anal verge to the level at which it turns into sigmoid colon), small bowel (2.5 cm beyond the PTV), and prostate were contoured on each scan. To eliminate inter‐observer variation, the same physician contoured the prostate in all patients. The PTV was generated by adding a 1‐cm three‐dimensional (3D) margin to the prostate. The length of the rectum was noted relative to the inferior set of fiducial markers.

The planning procedure was designed to follow the prostate radiation treatment protocol currently in use at our cancer center. Patients are aligned using skin tattoos, and portal images are taken for the first three fractions to verify alignment against bony landmarks.

In the present study, four plans were produced for every patient. These plans were each assigned a two‐letter acronym, the first letter indicating a CT scan to which radiation fields were applied; the second, the anatomy for which a multileaf collimator (MLC) pattern was produced. An E stands for empty bladder, and an F, for full bladder. For example, plan EE used empty‐bladder anatomy on CT, and the MLC pattern was specifically designed for that anatomy.

We first used dose–volume histograms (DVH) and biologic indices calculated for FF plans and for EE plans to consider the dosimetric consequences of treating on an empty bladder and on a full bladder. Second, we considered situations in which a patient planned on a full bladder comes for treatment with an empty bladder and vice versa. For example, the EF plan simulates a situation in which planning was based on full‐bladder anatomy, but the patient came for treatment with an empty bladder. The MLC pattern and the isocenter as derived from the full‐bladder scan were therefore applied to empty‐bladder treatment.

**Figure 2:**
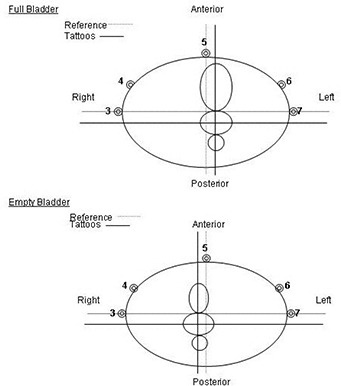
Illustration of isocenter location at prostate center of mass and of location of reference point at intersection of fiducial markers: full bladder (top) and empty bladder (bottom).

To facilitate comparison and interpretation of the treatment plans, we calculated prostate motion following voiding. For each scan, coordinates for the center of mass of the prostate were calculated. All coordinates were documented with reference to the intersection of lines drawn through fiducial markers 3, 5, and 7. Prostate motion in three dimensions was calculated as the difference in shifts of prostate center of mass relative to the calculated reference point (Fig. 2). To verify that the calculated prostate motion was not attributable to variation in prostate contouring, we checked prostate volumes in each patient for consistency. For 12 of 21 patients, the difference in prostate volume was less than 2 cm^3^. For all patients, the difference was less than 7 cm^3^.

Treatment planning was performed using a 3D planning system (Cadplan: Varian Medical Systems, Palo Alto, CA). A four‐field box technique with equally weighted 18‐MV beams at isocenter (gantry 0 degrees, 90 degrees, 180 degrees, 270 degrees) was used. For EE and FF plans, beams were centered on the prostate. The isocenter coordinates were therefore different because of the difference in the prostate center‐of‐mass position. Full‐ and empty‐bladder conformal plans were then generated using a 120 MLC system (Varian 21 EX Clinac). Dose calculations included a modified Batho correction to account for inhomogeneities, and plans were normalized to the center of the prostate—that is, the isocenter. The minimum dose to the PTV was 95% of the prescribed dose (7000 cGy in 35 fractions).

When the full‐bladder plan was placed onto the empty‐bladder anatomy (and vice versa), the intent was to simulate a situation in which a patient scanned and planned with a full bladder comes for treatment with bladder empty and receives the treatment planned from the full‐bladder scan. To place the isocenter, as noted above, the documented coordinates were used to place the field size and MLC shapes from the full‐bladder plan onto the empty‐bladder anatomy. Dose calculations were performed using the modified Batho correction and the normalization from the full‐bladder plan. The same number of monitor units (MUs) as calculated for the full‐bladder scan were applied to the empty‐bladder plan. A difference of 1 MU was deemed acceptable as being a result of rounding. On all plans, DVHs were calculated for the bladder, rectum, small bowel, prostate, and PTV, and these DVHs were exported for analysis.

### B. DVH analysis

The DVHs for rectum and bladder were reduced to single‐step histograms—that is, effective dose to the whole volume—using the power‐law method.^(11)^ The values of the parameter describing the strength of the volume effects, *n*, was set to 0.5 for bladder and 0.12 for rectum.^(^ ^12^ ^,^ ^13^ ^)^ The effective dose, $D_{\text{eff}}$, was calculated using the expression

(1) $$D_{eff}={\left(\sum D_{i}^{1/n}V_{i}\right)}^{n},$$

where $D_{i}$ is the dose to the partial volume $V_{i}$.

The biologic consequences of the dose distribution to prostate were evaluated using the equivalent uniform dose (EUD).^(^ ^14^ ^)^ The EUD requires two parameters: surviving fraction after delivery of 2 Gy $\left({\text{SF}}_{2}\right)$, which was set to 0.65(^15^); and α/β ratio, which was set to 1.5 Gy. Smaller ${\text{SF}}_{2}$ values—for example, ${\text{SF}}_{2}=0.5$—were used in model calculations accounting for tumor heterogeneity in radiosensitivity.^(^ ^16^ ^)^ In that case, tumor response is dominated by a radioresistant tail. Carlson et al.^(^ ^17^ ^)^ recently summarized linear‐quadratic radiosensitivity parameters reported in both *in vitro* and *in vivo* studies. If the *in vivo* parameters are translated to ${\text{SF}}_{2}$, values in the range 0.6–0.85 can be obtained, consistent with the ${\text{SF}}_{2}$ of 0.65 used in the present study. The NTCP was calculated using the Lyman–Kutcher–Burman formalism,^(^ ^11^ ^,^ ^12^ ^)^ with model parameters set to those reported in Burman et al.^(^ ^12^ ^)^

## III. RESULTS

**Figure 3:**
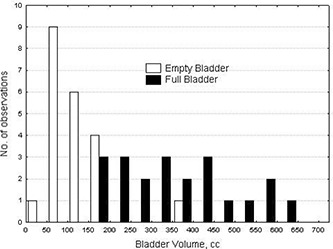
Distribution of bladder volume (empty and full bladder) for 21 patients.

Fig. 3 shows histograms for empty‐ and full‐bladder volumes in the study patients. Mean full‐bladder volume was 354.3 cm^3^ (range: $154.9-601.6\;{\text{cm}}^{3}$; median: 349.9 cm^3^), and mean empty‐bladder volume was 118.2 cm^3^ (range: $48.8-351.6\;{\text{cm}}^{3}$; median: 104.6 cm^3^). On average, bladder volume decreased by 236 cm^3^ after patients were allowed to void. Table 1 shows the full data for prostate, bladder, and rectum volumes. Notably, except for bladder volume, other organ volumes were not affected by the full or empty state of the bladder. That finding means that, for organs other than bladder, differences between dose distributions in EE and FE plans and in FF and EF plans can be attributable only to organ motion relative to external markers and not to changes in organ volumes. In contrast, for bladder, these differences—and particularly the differences between EE and FF plans—result from bladder volume changes.

**Table 1:** Mean volume (standard deviation) of considered organs with full and empty bladder, in cubic centimeters

	Bladder	Rectum	Prostate
Empty bladder	118.2 (67.9)	89.5 (37.7)	46.3 (20.7)
Full bladder	354.3 (140.5)	88.4 (34.3)	46.3 (20.8)

**Figure 4:**
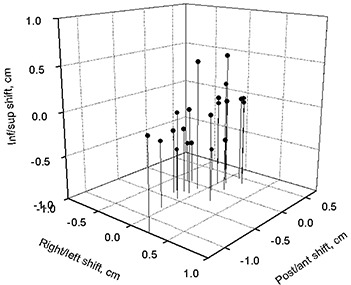
Three‐dimensional representation of prostate shifts following bladder voiding.

Prostate motion was examined. Fig. 4 shows a scatter graph for shifts in the position of the prostate center of mass in three dimensions. The prostate shifts were defined as change in the coordinates of the prostate center of mass relative to the external fiducial markers. The most pronounced shifts were in the superior–inferior and anterior–posterior dimensions. Table 2 further illustrates that finding.

**Table 2:** Prostate shifts following bladder voiding, in centimeters

Shift direction	Median	Mean	Standard deviation	Largest shift	
Left(+)/Right(−)	−0.04	−0.02	0.17	0.31 Left	0.25 Right
Anterior(+)/Posterior(−)	−0.01	−0.05	0.45	0.6 Anterior	1.24 Posterior
Superior(+)/Inferior(−)	−0.01	−0.10	0.30	0.50 Superior	0.52 Inferior

Although prostate motion was noted between full‐ and empty‐bladder scans for some patients, no systematic movement in any direction was observed. The largest shifts for specific patients were 1.24 cm in the posterior direction, 0.6 cm in the anterior, 0.52 cm in the inferior, and 0.50 cm in the superior. Of 21 patients, 10 showed prostate shifts in excess of 0.5 cm in one of the directions. On average, the shifts were insignificant, and no patient showed prostate shifts larger than 0.5 cm in more than one direction. Change in bladder volume did not correlate with prostate shifts; Table 3 shows the linear regression results. In contrast, rectal filling strongly correlated with prostate shifts in the anterior–posterior direction. Table 3 also shows those results.

**Table 3:** Correlation between prostate shift and changes in bladder and rectal volumes

Shift direction	$R^{2}$	*P*
Change in bladder volume		
Superior/inferior	0.034	0.423
Anterior/posterior	0.010	0.660
Lateral	0.012	0.633
Change in rectal volume		
Superior/inferior	0.034	0.425
Anterior/posterior	0.410	0.002
Lateral	0.076	0.225

Empty‐ (EE) and full‐bladder (FF) plans were both designed to ensure coverage of the PTV with the 95% isodose surface, with no dose–volume constraints applied for normal tissue. Median prostate EUD for the full‐bladder scans was 70 Gy (range: 69.7–70.5 Gy; mean: 70.0 Gy) as compared with 70 Gy (range: 69.7–70.6 Gy; mean: 70.1 Gy) for the empty‐bladder scans. That observation shows that the planning objective to contain the entire PTV within a 95% isodose surface was achieved equally well for both scans. Note that, to clearly identify and quantify the dosimetric consequences of bladder filling for normal tissues, no normal‐tissue constraints were applied in treatment planning.

**Figure 5:**
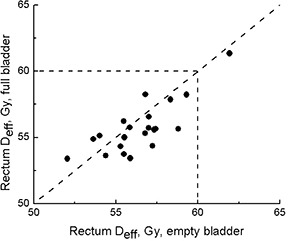
Effective dose $\left(D_{\text{eff}}\right)$ to rectum for empty‐ and full‐bladder computed tomography scans. Dashed lines indicate tolerance dose $\left({\text{TD}}_{5/5}\right)$ as reported by Emami et al.^(13)^

Fig. 5 shows effective rectum doses for full‐ (FF) and empty‐bladder (EE) scans. The median rectal $D_{\text{eff}}$ for the full‐bladder scans was 55.6 Gy (range: 53.4–61.4 Gy; mean: 55.7 Gy) as compared with 56.8 Gy (range: 52.0–61.9 Gy; mean: 56.5 Gy) for the empty‐bladder scans. Although more points fall below the 45‐degree line, indicating better rectal sparing for full‐bladder scans, the difference is marginal. The two lines drawn through the 60‐Gy points on both axes show the tolerance dose corresponding to 5% risk of complications, based on published data for tolerance dose $\left({\text{TD}}_{5/5}\right)$.^(13)^ For 1 patient, the rectum $D_{\text{eff}}$ was not contained within the area inside the dashed lines, indicating that the risk of rectal complications was excessive. An alternative plan was produced for this patient's actual treatment.

**Figure 6:**
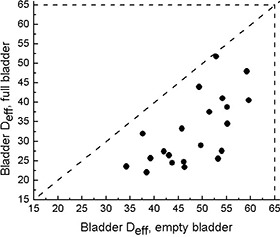
Effective dose $\left(D_{\text{eff}}\right)$ to bladder for empty‐ and full‐bladder computed tomography scans. Dashed lines indicate tolerance dose $\left({\text{TD}}_{5/5}\right)$ as reported in Emami et al.^(13)^

Fig. 6 presents the bladder $D_{\text{eff}}$ data. Expectedly, better bladder sparing was achieved when full‐bladder (FF) scans were used. For the full‐bladder scans, the median bladder $D_{\text{eff}}$ was 29.0 Gy (range: 22.0–51.8 Gy; mean: 32.4 Gy) as compared with 49.3 Gy (range: 34.2–59.6 Gy; mean: 48.1 Gy) for the empty‐bladder scans. For some patients, the change in effective dose was as high as 30 Gy. However, all the points were contained within the area set by the ${\text{TD}}_{5/5}$ lines,^(^ ^12^ ^,^ ^13^ ^)^ and the NTCP values did not exceed 2% for either group.

**Figure 7:**
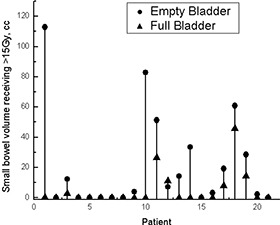
Small bowel volumes receiving more than 15 Gy for empty‐ and full‐bladder computed tomography scans.

Because only a portion of small bowel was contoured, dose–volume data for the whole organ are not available. Fig. 7 shows absolute volumes receiving more than 15 Gy.^(18)^ In 8 empty‐bladder (EE) and 15 full‐bladder (FF) scans, no part of the small bowel received more than 15 Gy. Better small‐bowel sparing can be achieved if full‐bladder scans are used; however, the absolute volume of small bowel receiving doses greater than 15 Gy is small—less than 120 cm^3^ in all cases. If a 50‐Gy cutoff dose had been applied,^(^ ^13^ ^,^ ^19^ ^)^ part of the small bowel would have received more than 50 Gy in 1 patient with a full bladder (7.5 cm^3^), and in 6 with an empty bladder $\left(2.5\;{\text{cm}}^{3}-30\;{\text{cm}}^{3}\right)$. The largest volume receiving more than 50 Gy was only 30 cm^3^.

**Figure 8:**
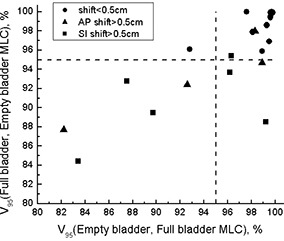
Planning target volumes (*V*s) receiving at least 95% of the prescription dose. As planned, $V_{95}=100\%$. MLC=multileaf collimator; AP=anterior–posterior; SI=superior–inferior.

Fig. 8 shows how PTV coverage is affected if the MLC shape produced for an empty‐bladder scan is applied to full‐bladder anatomy (FE) and vice versa (EF). The dashed lines indicate $V_{95}$—that is, 95% of the PTV receiving 95% of the prescribed dose. As planned, $V_{95}=100\%$ by design, with the MLC adjusted to tightly conform to the PTV so as to minimize the dose to the surrounding normal tissues. For patients with a prostate shift larger than 0.5 cm relative to external fiducial markers, the PTV can be severely underdosed. Notably, all 5 patients receiving less than 95% of prescription dose to less than 95% of the PTV had prostate shifts larger than 0.5 cm. For patients who showed small shifts relative to fiducial markers, underdosing of the PTV, if any, was also small. In terms of PTV coverage, FE and EF plans were equally detrimental. Although Fig. 8 uses the terms “empty” and “full” bladder, it was not bladder filling that caused the prostate (and consequent PTV) shifts. These findings are therefore more reflective of a change in anatomy within approximately 1 hour.

**Figure 9:**
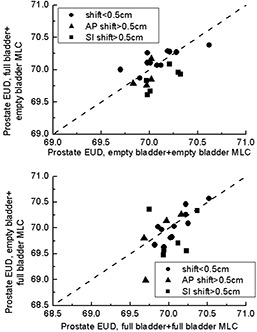
Prostate equivalent uniform dose (EUD) for situations in which a multileaf collimator (MLC) pattern designed for one scan was applied to a different scan. AP=anterior–posterior; SI=superior–inferior.

For patients with a large prostate shift, the PTV coverage was compromised; however, underdosing of prostate itself did not result (Fig. 9). If the EUD did not change at all, the point would fall on the dashed line. Points below this line indicate patients who will receive an EUD below that planned. The upper panel shows how the EUD would change if a patient planned on an empty bladder came for treatment with a full bladder (FE). None of the patients showed an EUD below 69.5 Gy for an empty‐bladder MLC applied to full‐bladder anatomy. For the opposite (EF) situation, 1 patient showed an EUD of 69 Gy. However, that patient's planning EUD was 69.7 Gy. Overall, prostate coverage is acceptable, which proves that the PTV margins used are adequate even for large shifts.

**Figure 10:**
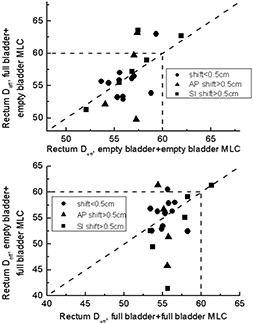
Rectal effective dose $\left(D_{\text{eff}}\right)$ for situations in which a multileaf collimator (MLC) pattern designed for one scan was applied to a different scan. Dashed lines indicate tolerance dose $\left({\text{TD}}_{5/5}\right)$ as reported in Emami et al.^(13)^ AP=anterior–posterior; SI=superior–inferior.

Fig. 10 shows how the effective dose to rectum changes in FE (upper panel) and EF (lower panel) plans. The dashed lines show the ${\text{TD}}_{5/5}$ for the whole organ.^(13)^ For 1 patient, the planning $D_{\text{eff}}$ exceeded acceptable dose–volume constraints. In clinical practice, this plan would have been rejected, and an alternative beam arrangement would have been considered. On 5 occasions, patients whose planning $D_{\text{eff}}$ had been less than 60 Gy moved above the 60 Gy threshold. Although the largest changes in $D_{\text{eff}}$ were seen for patients with prostate shifts larger than 0.5 cm in one of three directions, a large shift is not prerequisite. On 2 of 5 occasions when the 60‐Gy $D_{\text{eff}}$ threshold was exceeded, the shift was less than 0.5 cm.

**Figure 11:**
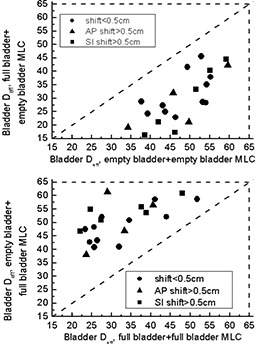
Bladder effective dose $\left(D_{\text{eff}}\right)$ for situations in which a multileaf collimator (MLC) pattern designed for one scan was applied to a different scan. Dashed lines indicate tolerance dose $\left({\text{TD}}_{5/5}\right)$ as reported in Emami et al.^(13)^ AP=anterior–posterior; SI=superior–inferior.

Fig. 11 shows how bladder $D_{\text{eff}}$ would change if a patient who was planned on an empty bladder were to be treated with a full bladder (FE plan, upper panel) and vice versa (EF plan, lower panel). This case is the only one in which a change in dose–volume statistics can be related to organ change rather than to changes in anatomy not related to bladder filling (as appears to be the case for prostate and rectum). As expected, for patients who were planned on an empty bladder, better bladder sparing is achieved if the patient arrives for treatment with a full bladder. On the other hand, substantial detrimental change in $D_{\text{eff}}$ may occur if a patient planned with a full bladder arrives for treatment with an empty bladder. The lower panel in Fig. 11 shows that, for some patients, the effective dose changes by as much as 30 Gy, although the ${\text{TD}}_{5/5}$ ^(13)^ is never exceeded.

## IV. DISCUSSION

In the present study, we looked at the impact of bladder filling on prostate radiotherapy dosimetry. Although the patients were given clear instructions how to prepare for the full‐bladder and empty‐bladder scans, the range of bladder volumes in both series was very wide, indicating significant variation in individual bladder volume and possibly in individual perception of bladder fullness. Prostate shifts are apparent between the full‐bladder and empty‐bladder scans. Magnitude of the prostate shifts is consistent with the results from other institutions.^(^ ^2^ ^–^ ^5^ ^)^ In most cases, magnitude of the prostate shift relative to the external fiducial markers is small (the mean shifts being less than 0.1 cm in any direction), but the shift can exceed 0.5 cm for a specific patient. Prostate motion showed no consistent correlation with bladder filling in the present study.

Prostate EUDs were not significantly different when planned either on a full‐bladder scan or an empty‐bladder scan. That finding is not unexpected, because the radiotherapy plan is designed to encompass the PTV optimally, whether the planning CT is done with a full or empty bladder. The rectal $D_{\text{eff}}$ was also not affected by bladder state, full or empty, during the acquisition of the planning CT. That finding implies that the *irradiated* rectal volumes are not significantly affected by bladder filling, although the possibility that rectal shape and size (potentially affected by rectal peristalsis) can be different between the empty‐ and full‐bladder scans is not excluded.

Although doses to the small bowel may be slightly higher in the empty‐bladder dosimetry, they are within tolerance.^(^ ^13^ ^,^ ^19^ ^)^ Letschert et al.^(19)^ reported a correlation between small‐bowel volume receiving 50 Gy or higher and bowel complications. The actuarial 5‐year estimate of chronic diarrhea varied from 31% in patients with irradiated small‐bowel volumes below 77 cm^3^ to 42% in patients with irradiated volumes above 328 cm^3^. In the present study, the maximum small‐bowel volume receiving more than 50 Gy was just 30 cm^3^.

Our results clearly demonstrate that bladder sparing is indeed significantly improved for patients treated on a full bladder. However, bladder complication rates are fairly low, with an estimated NTCP of less than 2% in the full‐bladder and empty‐bladder cases. In institutions in which an empty bladder is used as part of the prostate radiotherapy protocol,^(^ ^6^ ^,^ ^7^ ^)^ the published genitourinary (GU) toxicity has been low and comparable with results from other institutions.

The key question is not whether DVHs for a full bladder are superior to those for an empty bladder, but rather whether the empty‐bladder DVHs are clinically acceptable. If the RTOG P0126 guidelines are followed, then constraints on bladder DVHs must obey these requirements: $V_{80}<15\%,\;V_{75}<25\%,\;V_{70}<35\%,\;and\;V_{65}<50\%$, where $V_{x}$ is the percentage volume receiving a dose of at least *x* Gy. Because the prescription dose is 70 Gy, only constraints for doses 70 Gy or less could have been violated. Of the 21 patients involved in the present study, the EE plans for only 2 showed DVHs that did not obey the $D_{65}$ constraint.

Although bladder DVH guidelines^(^ ^6^ ^,^ ^7^ ^)^ have been suggested (RTOG P0126), most of the published results do not support a correlation between bladder DVH and GU toxicity.^(^ ^20^ ^–^ ^24^ ^)^ W e suspect that, although bladder dose is related to GU toxicity, the critical organs may be the bladder neck itself and the urethra, which often are not analyzed in external‐beam radiotherapy data. In addition to the known daily variation of bladder filling, bladder DVH alone may not be the ideal factor for GU toxicity correlation. The combination of bladder and bladder neck DVH with urethral DVH is likely to be a better model in correlations of GU toxicity.

The presented data for MLC pattern, when applied in reverse, show an extreme case when the MLC pattern designed for one scan is applied to a different scan throughout treatment. In reality, if a patient is planned with bladder full, but is not able to maintain fullness throughout treatment, the process will be very gradual. Therefore, the data presented in Fig. 11 have to be treated as the worst‐case scenario.

A systematic reduction in bladder volume through the course of prostate radiation therapy has been observed clinically. Zellars et al.^(1)^ reported that bladder volume in 24 prostate patients treated supine with a full‐bladder protocol decreased to 51% of pretreatment volume after 4–5 weeks of radiation therapy.

Although the $D_{\text{eff}}$ threshold corresponding to ${\text{TD}}_{5/5}$ was never exceeded for the patients considered in the present study, the proposed RTOG P0126 DVH restrictions are in some cases violated. Specifically, for the patient who showed a change in bladder $D_{\text{eff}}$ from 30 Gy to 64 Gy, the $D_{65}$—that is, the bladder volume receiving more than 65 Gy—was 74%, well in excess of the less than 50% in the RTOG guidelines. Notably, this patient showed a very large change in bladder volume—from 419 cm^3^ to 90 cm^3^. Conceivably, some patients may experience significant changes in bladder volume (for example, because of urinary symptoms), thereby putting them at risk of bladder complications.

Changes in dose distributions in organs and volumes other than bladder have to be treated with care. Although we referred to scans or MLC patterns as “full‐bladder” and “empty‐bladder,” organs outside of the bladder showed no directional motion or change of volume because of bladder filling. In patients who showed shifts in prostate location larger than 0.5 cm in any one direction, PTV coverage was compromised. However, compromised dose delivery to the prostate did not result.

Although change in rectal dose distribution cannot be attributed to bladder filling, such change is quite marked. No directional preference was noted, whether an empty‐bladder MLC pattern was applied to full‐bladder anatomy or vice versa: rectal $D_{\text{eff}}$ was reduced in some patients and increased in others. Patients with large prostate shifts also tended to show larger changes in rectal $D_{\text{eff}}$; nevertheless, 1 patient who showed no shift in excess of 0.5 cm showed a significantly changed rectal dose distribution when his empty‐bladder MLC pattern was applied to his full‐bladder anatomy. If RTOG guidelines for rectal DVH were to be applied, then the plan would not have been acceptable. This patient's $V_{60}$—that is, partial rectal volume receiving at least 60 Gy—changed from 34% to 48% when the empty‐bladder MLC pattern was applied to the full‐bladder scan. The RTOG guideline is less than 50%. Similarly, $V_{65}$ changed from 27% to 43%, in violation of the 35% guideline, and $V_{70}$ changed from 7% to 23%, close to violating 25% guideline. This finding shows that, within a span of 1 hour, patient anatomy can change to the extent that a plan deemed to obey normal‐tissue tolerance guidelines no longer complies with those guidelines, reinforcing the possibility that significant geometric errors, in particular systematic errors, may put patients at increased risk of complications.

## V. CONCLUSIONS

Bladder filling has no significant impact on prostate EUD or rectal $D_{\text{eff}}$. In both full‐ and empty‐bladder scans, only a minimal volume of small bowel received more than 50 Gy, which is below dose tolerance. The bladder $D_{\text{eff}}$ is higher with an empty bladder; however, with the currently available NTCP data, the predicted complication rates are clinically insignificant. Substantial changes in dose distributions were noted if, with beams aligned to external fiducial markers, the MLC pattern designed for an empty‐bladder scan was applied to full‐bladder anatomy and vice versa. Substantial PTV underdosing was shown. The underdosing was particularly pronounced for patients with prostate shifts in excess of 0.5 cm in any single direction relative to external fiducial markers. Dose to the prostate itself was not compromised. Changes in dose distributions to the prostate and rectum in EF and FE plans cannot be correlated with bladder filling. For the EF and FE plans, dose distributions in rectum and, particularly, in bladder change substantially. For some patients, in the worst‐case scenario, bladder tolerance may be exceeded for the EF plan—that is, when a patient planned on a full bladder arrives for treatment with an empty bladder throughout treatment.
